# Effect of the Algicide Thiazolidinedione 49 on Immune Responses of Bay Scallop *Argopecten Irradians*

**DOI:** 10.3390/molecules24193579

**Published:** 2019-10-04

**Authors:** Cheng Chi, Saekil Yun, Sib Sankar Giri, Hyoun Joong Kim, Sang Wha Kim, Jeong Woo Kang, Se Chang Park

**Affiliations:** 1 Key Laboratory of Aquatic Nutrition and Feed Science of Jiangsu Province, National Experimental Teaching Center for Animal Science, College of Animal Science and Technology, Nanjing Agricultural University, Weigang Road 1, Nanjing 210095, China; chicheng0421@126.com; 2 Laboratory of Aquatic Biomedicine, College of Veterinary Medicine and Research Institute for Veterinary Science, Seoul National University, Seoul 151–747, Korea

**Keywords:** algaecide, thiazolidinedione 49, *Argopecten irradians*, immune response

## Abstract

The thiazolidinedione 49 (TD_49_) is an effective algaecide against harmful algae; however, its potential effects on the immune function of the edible bay scallop are unclear. Therefore, the present work studied the effects of TD_49_ on the immune response in bay scallop by evaluating activities of acid phosphatase (ACP), alkaline phosphatase (ALP), and superoxide dismutase (SOD), as well as nitric oxide (NO) levels, total protein content, and expression of immune genes (*CTL-6*, *PGRP*, *PrxV*, *MT*, and *Cu/Zn-SOD*) at 3–48 h post-exposure (hpe) to TD_49_. The activities of ACP and ALP significantly increased in TD_49_-treated groups at 3–24 hpe, whereas NO levels decreased significantly in 0.58 and 0.68 μM of TD_49_ at 6–24 hpe, after which the level was similar to that in the untreated control. Moreover, SOD activity significantly increased in all three concentration groups at 3–6 hpe, while it decreased at 12 hpe in the 0.68 μM TD_49_ treatment group. Notably, total protein content increased with TD_49_ treatment at each time interval. The results revealed that variable effects on the expression of immune-related genes were observed after treatment with TD_49_. The findings demonstrate that exposure of scallops to TD_49_ changes immune responses and expression of immune-related genes. We hypothesize that TD_49_ may disrupt immune system in bay scallop. The current investigation highlights the potential negative effects of using TD_49_ as an algaecide on marine economic bivalves to control harmful algal blooms in marine environments.

## 1. Introduction

In coastal waters, harmful algal blooms (HABs) have increased rapidly with increasing eutrophication and changing environmental conditions. HABs could cause a variety of issues in aquatic ecological systems, the fishery industry, and may even threaten human public health [1]. Many researchers have studied physiological and ecological methods to control and reduce the loss for fisheries caused by HABs [2,3]. Physical methods, such as clay, have been used extensively for their low cost and the ease of use; however, clay may adhere to the gills of aquatic organisms, increasing morbidity and mortality. Other methods, including hydrogen peroxide, simazine, copper sulphate, potassium permanganate, and viral biological agents, have also been demonstrated to cause ecological and environmental stresses in non-target species. Therefore, developing environmentally friendly algaecides is important. Thiazolidinedione (TD) was introduced in the late 1990s as an adjunctive therapy for the treatment of diabetes. TDs act by binding to peroxisome proliferator-activated receptors, a group of receptor molecules inside the cell nucleus [1] and have been very effective and selective for the HABs studied, with half-maximal inhibitory concentrations (IC_50_s) in the nanomolar range [2]. TD_49_ is a newly synthesized algaecide with strong toxicity at 0.1–2 μM for harmful algae, including *Heterosigma akashiwo*, *Chattonella marina*, and *Cochlodium polykrikoides*, while it has quite low toxicities for nonharmful algae, even at concentrations above 100 μM [2].

The new International Organization for Standardization (ISO) standard method, which uses *Ulva pertusa* Kjellman, as well as three marine representative species including algae, fish, and crustaceans, is usually employed for acute toxicity assessment. A previous study reported that the acute toxicity assessment using *Skeletonema costatum*, *Paralichthys olivaceus*, and *Daphnia magna* showed the LC_50_ (or EC_50_) of TD_49_ for the three species was 0.34, 0.58, and 0.68 μM, respectively [1]. However, to comprehensively assess the algaecide, the acute-toxicity assessment in these three species is insufficient. The low-toxicity algaecide should not only be less toxic to the representative species, but also should have minimal effects on non-target species, especially for some economically important aquatic species.

Therefore, to develop a novel environmentally friendly algaecide, its potential effects on non-target species must be determined and clarified. Li et al. [3] reported that palmitoleic acid (PA), isolated and identified from the metabolites of microorganisms, was active against *Alexandrium tamarense*. However, in their study, there were no data to demonstrate that PA was nontoxic or less toxic. Thus, our previous studies investigated the potential effects of PA on *Argopecten irradians*, which is an important economic aquatic species in South Korea and China. We studied the effects of PA on multiple physiological parameters of scallops, including the activities of superoxide dismutase (SOD), lactate dehydrogenase (LDH), alkaline phosphatase (ALP), acid phosphatase (ACP), and lysozymes. We also measured contents of nitric oxide (NO), glutathione (GSH), and malondialdehyde (MDA), which are related to immune, stress, and detoxification responses and have been measured in our previous studies [4,5]. We accordingly revealed that PA produced stress responses, induced oxidative stress, and disturbed immune responses in bay scallops [4,5,6]. Hence, before wide use of a novel algaecide, its potentially negative effects or physiological responses it may produce in non-target species, particularly those of economic importance, must be clarified.

Bivalve molluscs, including scallops, are economically important species in coastal areas of China, Korea, and Japan. Given their sessile nature, filter-feeding habits, and pollutant concentrations, they are also widely used in toxicology investigations and as marine pollution indicators [7]. Scallops are usually commercially cultured in shallow water by the rope-growing method, and therefore are vulnerable to environmental contaminants. Therefore, scallops are useful as bioindicators for monitoring studies [7,8]. Scallops lack an acquired immune system, so they only rely on their innate immune mechanisms to overcome the impacts of pathogenic invasions and diseases [9]. The nonspecific immune system of bivalves depends on circulating cells and a series of molecular effectors. Biologically active molecules in scallop hemolymph are classified into two categories: Lysosomal enzymes (esterases, acid phosphatase, alkaline phosphatase, peroxidases aminopeptidases, β-glucuronidases, and α-mannosidase) and serologically active agents (antimicrobial factors, lysins, opsonins, agglutinins, lysozymes) [9]. ALP, which is a polyfunctional phosphomonoester hydrolase, is a short-term biomarker for stress [10]. In addition, some antimicrobial compounds, such as ACP, are involved in humoral immune responses and participate in the degradation of foreign carbohydrates, proteins, and lipids [4]. The inducible nitric oxide (NO) synthesized by inducible (i) NO synthase (iNOS) is usually considered to be related to the immune response in mammals. In bivalves, NO has been reported to be involved in innate immunity, iNOS-like activity has been detected in hemocytes of the zhikong scallop *Chlamys farreri* after LPS stimulation [11,12], and NO has been shown be synthesized in scallop hemocytes [13], directly or indirectly contributing to pathogen elimination. It has been demonstrated that NO promotes apoptosis and enhances phagocytic and anti-bacterial ability by developing oxidative toxicity while avoiding excessive auto-toxicity during the last phase of immunity, playing a crucial role in the modulation of the immune response [11,12]. SOD, as an antioxidating enzyme, is considered to be the first line of defense against reactive oxygen species (ROS) to protect tissues from oxidative damage. Therefore, monitoring of NO, ALP, ACP, and SOD is an essential component for understanding the nonspecific immune responses of scallops [5,14]. In addition, our previous study revealed that, following 0.68 μM TD_49_ exposure for up to 48 h, gene expressions in the gills of bay scallop related to ATP-binding cassette, nuclear factor erythroid 2-related factor, B cell lymphoma-2 family protein, glutathione reductase, glutathione peroxidase, catalase, NADPH2:quinone reductase, and superoxide dismutase were decreased. Conversely, gene expressions related to FAS-associated death domain protein, glutathione S-transferase, caspase 6, 8, cytochrome P450 1A1, and 2C8 were increased. These results comprehensively demonstrated the toxicity of the novel algicide TD_49_ [15]. However, the previous investigation only revealed the potential impact of TD_49_ on transcriptional level, the immunotoxicity of TD_49_ on bay scallop should draw the attention of researchers to study further.

Therefore, the current investigation was designed to evaluate and quantify the effects of TD_49_ on the immune responses induced in scallops in the context of the new ISO standard toxicity assessment method. These results, as a supplement to the comprehensive toxicity assessment of the novel algaecide TD_49_, will subserve to provide guidelines for the establishment of standards for the development of algaecides with a relatively benign effect on marine ecology, especially coastal economical species.

## 2. Results

### 2.1. TD_49_ Treatment Alters Enzymatic Activities Important for Nonspecific Immune Responses

**Figure 1:**
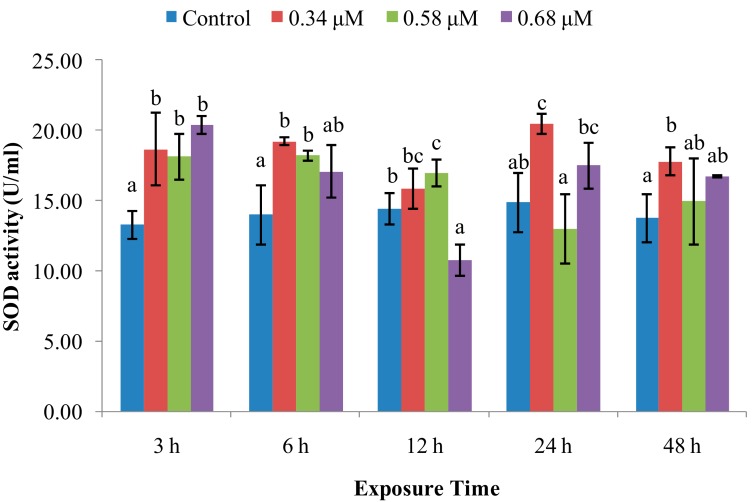
Effect of TD_49_ on hemolymph superoxide dismutase (SOD) activity at different time points after exposure to different concentrations (0, 0.34, 0.58, 0.68 μM) of TD_49_. Data represent mean ± SD values (*n* = 3) at the same time interval, with different letters denoting significant differences (*p <* 0.05).

SOD activity increased in scallops treated with 0.34 μM of TD_49_ after 3–48 h post-exposure (hpe), except at 12 hpe (Figure 1). The SOD activity in the 0.58 μM treatment group significantly increased at 3–12 hpe, returning to normal levels at 24 and 48 h. However, SOD activity in the 0.68 μM treatment group significantly increased only at 3 hpe, and then decreased transiently at 12 hpe. Thereafter, the activity returned to normal levels at 24–48 hpe.

**Figure 2:**
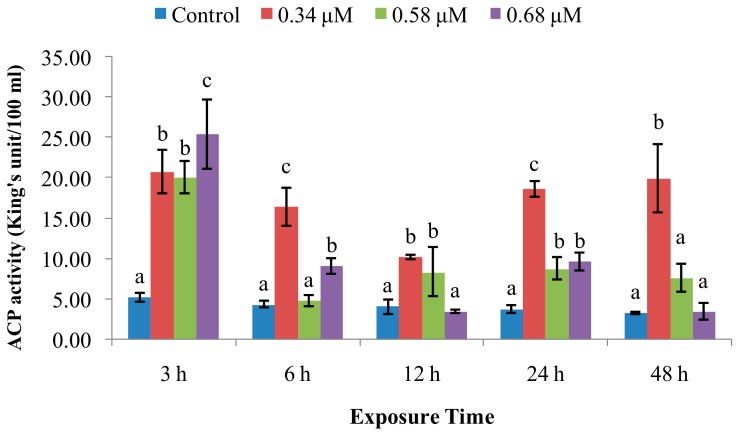
Effect of TD_49_ on hemolymph acid phosphatase (ACP) activity at different time points after exposure to different concentrations (0, 0.34, 0.58, 0.68 μM) of TD_49_. Data represent mean ± SD values (*n* = 3) at the same time interval, with different letters denoting significant differences (*p* < 0.05).

ACP activity (Figure 2) was significantly increased at 0.34 μM of TD_49_ at all time points. In the group treated with 0.58 μM, ACP activity was significantly increased at 3 hpe, no significant change at 6 hpe, then increased at 12 and 24 hpe, finally returning to normal at 48 hpe. ACP activities for the 0.68 μM group also fluctuated, increasing at 3 and 6 hpe, decreasing to normal at 12 hpe, increasing at 24 hpe, and decreasing to control levels at 48 hpe. 

**Figure 3:**
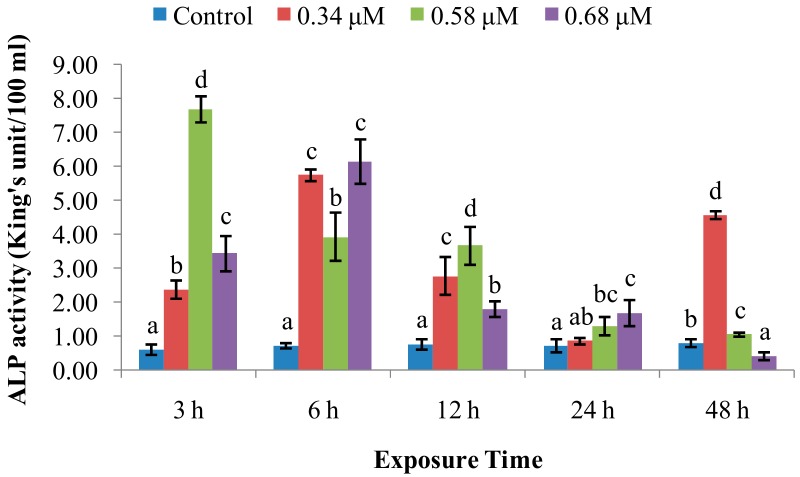
Effect of TD_49_ on hemolymph alkaline phosphatase (ALP) activity at different time points after exposure to different concentrations (0, 0.34, 0.58, 0.68 μM) of TD_49_. Data represent mean ± SD values (*n* = 3) at the same time interval, with different letters denoting significant differences (*p* < 0.05).

ALP activity (Figure 3) was significantly increased over control values at 3–12 hpe. Thereafter, it returned to near-normal levels at 24 and 48 hpe with one exception (0.34 µM), which was high at 48 hpe. Peak ALP activities for the 0.58 μM and 0.68 μM groups were reached at 3 and 6 hpe, respectively.

**Figure 4:**
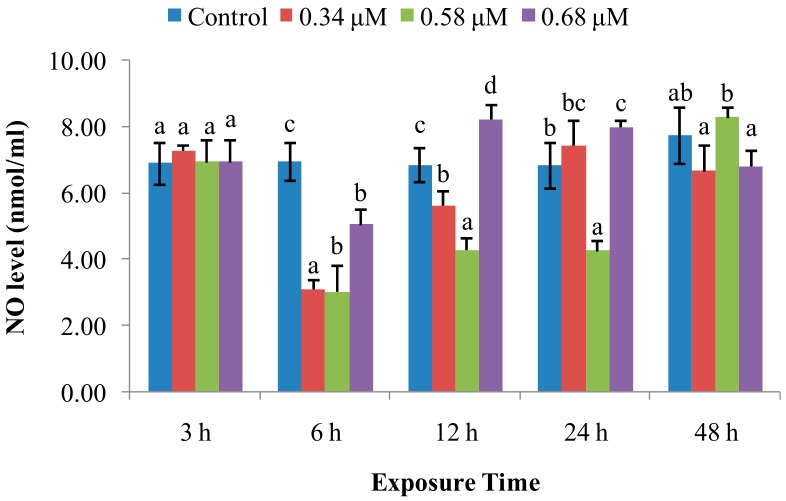
Effect of TD_49_ on nitric oxide (NO) levels at different time points after exposure to different concentrations (0, 0.34, 0.58, 0.68 μM) of TD_49_. Data represent mean ± SD values (*n* = 3) at the same time interval, with different letters denoting significant differences (*p* < 0.05).

NO levels (Figure 4) were unchanged at the 3 hpe time point. After 3 hpe, NO levels for the 0.34 and 0.68 μM groups sharply decreased, with one exception, at 6 and 12 hpe. Thereafter, levels returned to near-normal values at 24 and 48 hpe. Similarly, NO level in the 0.58 μM group significantly decreased at 6–24, and then returned to the normal level up to 48 hpe.

**Figure 5:**
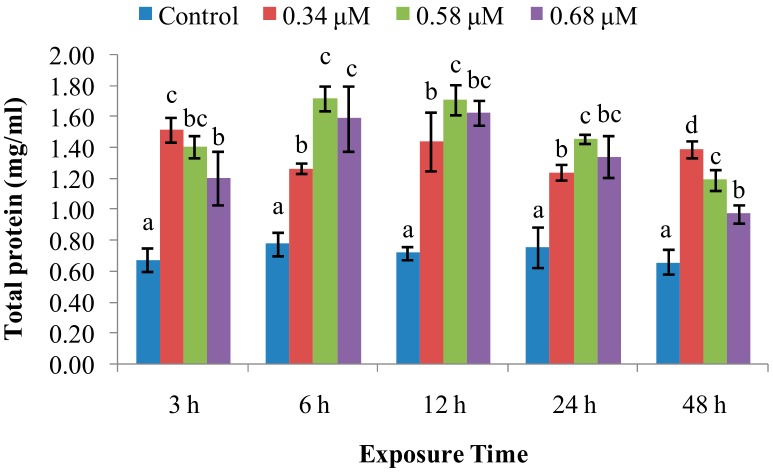
Effect of TD_49_ on total protein levels in hemolymph at different time points after exposure to different concentrations (0, 0.34, 0.58, 0.68 μM) of TD_49_. Data represent mean ± SD values (*n* = 3) at the same time interval, with different letters denoting significant differences (*p* < 0.05).

Total protein levels (Figure 5) were increased (*p* < 0.05) by all three concentrations of TD_49_ tested over the untreated control group.

### 2.2. Expression of Immune System-Related Genes

**Figure 6:**
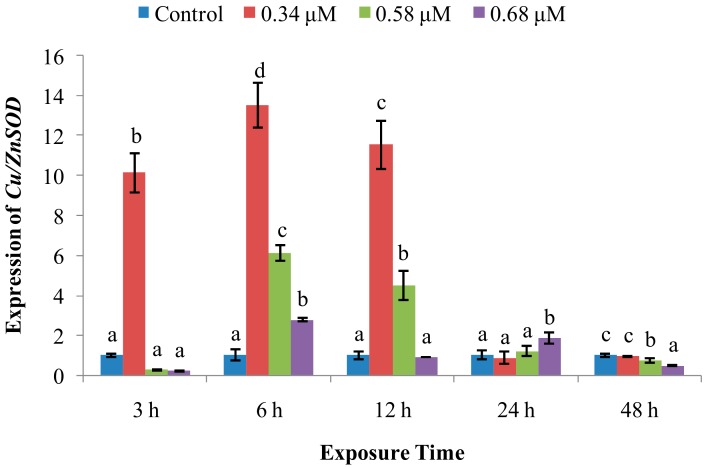
Effect of TD_49_ on hemolymph *Cu/ZnSOD* expression at different time points after exposure to different concentrations (0, 0.34, 0.58, 0.68 μM) of TD_49_. Data represent mean ± SD values (*n* = 3) at the same time interval, with different letters denoting significant differences (*p* < 0.05).

The expression of *CuZn-SOD* (Figure 6) was significantly increased by exposure to 0.34 μM of TD_49_ at 3, 6, and 12 hpe, returning to normal at 24–48 hpe, while we observed a more gradual increase for the 0.58 μM treatment group expression at 6 and 12 hpe. However, expression in the 0.68 μM treatment group was indistinguishable from that in controls at all time points.

**Figure 7:**
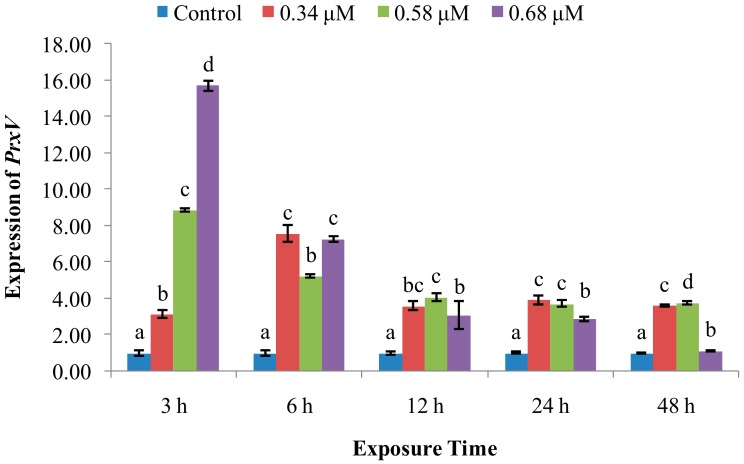
Effect of TD_49_ on hemolymph expression of *PrxV* at different time points after exposure to different concentrations (0, 0.34, 0.58, 0.68 μM) of TD_49_. Data represent mean ± SD values (*n* = 3) at the same time interval, with different letters denoting significant differences (*p* < 0.05).

The expression of *PrxV* (Figure 7) was significantly higher than control expression throughout the time course of treatment at all three TD_49_ concentrations, with the highest values observed at 6 hpe in the 0.34 μM treatment group and at 3 hpe in the 0.58 and 0.68 µM groups. Thereafter, the expression tended to decline up to 48 hpe.

**Figure 8:**
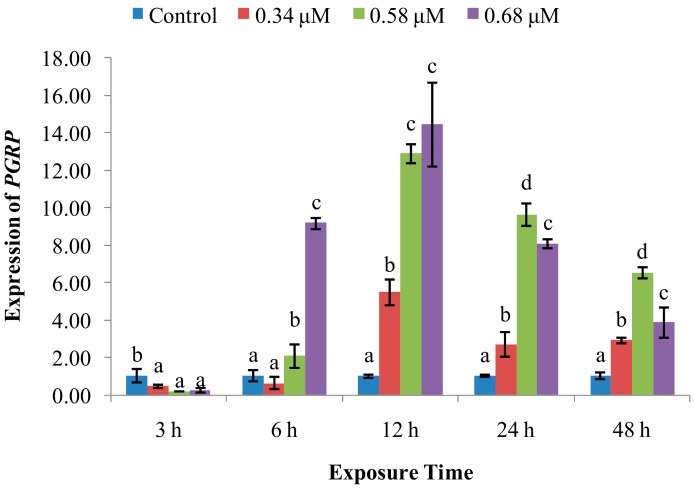
Effect of TD_49_ on hemolymph expression of *PGRP* at different time points after exposure to different concentrations (0, 0.34, 0.58, 0.68 μM) of TD_49_. Data represent mean ± SD values (*n* = 3) at the same time interval, with different letters denoting significant differences (*p* < 0.05).

*PGRP* mRNA expression (Figure 8) was decreased at 3 hpe, after which it increased at 6 and 12 hpe as an apparent function of TD_49_ concentration. At 24 and 48 hpe, *PGRP* expression progressively decreased, but remained elevated over control levels.

**Figure 9:**
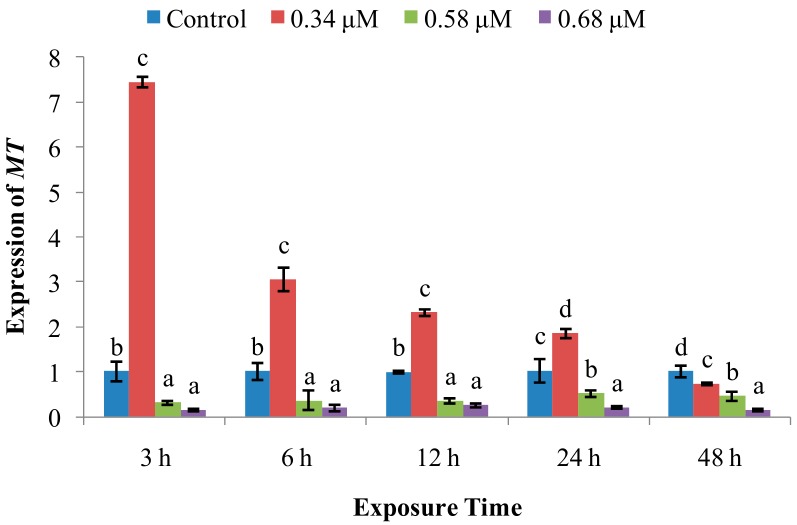
Effect of TD_49_ on hemolymph expression of *MT* at different time points after exposure to different concentrations (0, 0.34, 0.58, 0.68 μM) of TD_49_. Data represent mean ± SD values (*n* = 3) at the same time interval, with different letters denoting significant differences (*p* < 0.05).

Scallops treated with 0.34 μM of TD_49_ exhibited sharply higher *MT* expression (Figure 9) than that in control at 3 hpe, remaining higher through 24 hpe, gradually decreasing. By 48 hpe, levels were below control levels. Interestingly, the two higher concentrations decreased *MT* transcript levels at all five time points.

**Figure 10:**
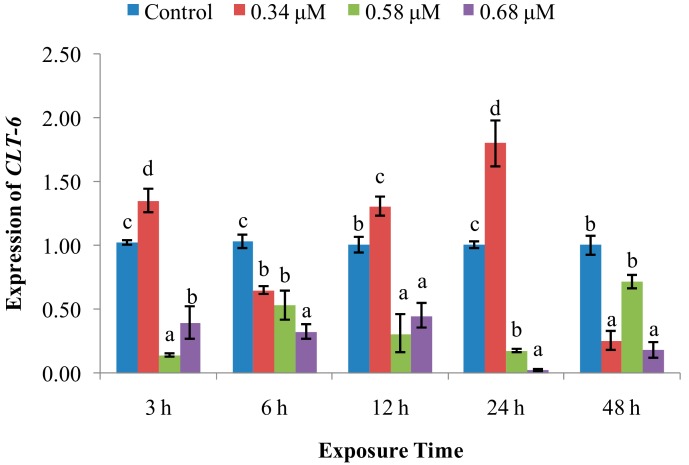
Effect of TD_49_ on hemolymph expression of *CLT-6* at different time points after exposure to different concentrations (0, 0.34, 0.58, 0.68 μM) of TD_49_. Data represent mean ± SD values (*n* = 3) at the same time interval, with different letters denoting significant differences (*p* < 0.05).

*CTL-6* mRNA expression (Figure 10) in the 0.34 μM group undulated over time. However, the *CTL-6* expression in the 0.58 and 0.68 μM groups decreased at 3–48 hpe, compared with control.

## 3. Discussion

An effective immune response is indispensable for growth, reproduction, and survival [16,17]. However, numerous reports document the vulnerability of an organism’s immune system to environmental exogenous stimuli, including algaecides used for controlling harmful algae blooms [4,5,6,18,19]. Previous studies revealed that exposure to a novel algaecide palmitoleic acid (PA) induced oxidative stress, immunotoxicity, and disrupted metabolism in bay scallops [4,5,6]. The promising new algaecide TD_49_ shows strong toxicities against harmful red-tide algal species at low concentrations of 0.1 to 2 μM, with low toxicities for nonharmful algae, even at over 100 μM [1,2]. Our results evaluated how exposure to sub-lethal concentrations of TD_49_ alters nonspecific immune responses in the bay scallop *A. Irradians*.

SOD is a metalloenzyme that plays an important role in the defence against ROS, removing O_2_^−^, preventing generation of highly toxic OH^−^, and catalyzing the dismutation of superoxide radicals into molecular oxygen and hydrogen peroxide [20,21]. In this study, SOD activity was increased at initial 3–12 hpe, with increasing *CuZn-SOD* expression at 3–12 hpe, and then returned to control levels at 12, 24, and 48 hpe in the low and medium concentration of TD_49_ treatment groups. These data suggest that the rapid increase of SOD activity and *CuZn-SOD* expression rapidly occurred to prevent oxidation induced by TD_49_ exposure. Our previous investigation also revealed that the *CuZn-SOD* expression in the gills of bay scallop decreased after 0.68 μM of TD_49_ exposure up to 48 hpe by transcriptomics analysis [15]. These results are consistent with the previous investigation that showed increased SOD activity in hemolymph of bay scallops after exposure to the algaecide PA [4]. The current results are also consistent with a previous study showing that at low benzo(*a*)pyrene (BaP) concentrations, the SOD activity of hemocytes increased initially, then decreased nearly to control levels [22]. However, the decrease in SOD activity observed in the high-concentration treatment group at 12 hpe suggests an inhibition of SOD activity and subsequent induction of oxidative stress, with SOD activities for benzo(k)fluoranthene (BKF) or a mixture of polycyclic aromatic hydrocarbons (PAHs) enhanced during the exposure time, while at higher concentrations all initially increased, then decreased [22]. Peroxiredoxin V (PrxV) is essential in the prevention of oxygen stress; this important role in maintaining cellular structural and functional integrity has been supported by many other studies [23]. Human PrxV has been shown to protect mitochondrial DNA from damage induced by H_2_O_2_ [23]. In the current research, the expression of *PrxV* increased in all time points after TD_49_ exposure, which indicates that increasing expression of *PrxV* was induced to prevent the oxidative stresses caused by TD_49_ exposure. However, the downward trend of *PrxV* expression with the extension of exposure time suggests that a long exposure time may decrease the *PrxV* expression, subsequently reducing the antioxidant ability of bay scallop. The antioxidant system involving SOD activity, expression of *CuZn-SOD* and *PrxV* was induced to synergistically resist to TD_49_ exposure. NO is a crucial signaling molecule that plays an irreplaceable role in the invertebrate immune system [11]; its modulation is important for maintaining immune homeostasis and a variety of normal physiological processes [11]. Here, we found that NO levels were decreased in the hemolymph of bay scallops treated with low and medium concentrations of TD_49_ at 6 and 12 hpe, as well as for the high concentration at 6 hpe. The phenomenon might be due to the elevated *PrxV* expression, since it was demonstrated that the antioxidant response, dependent on PrxV, acts as a negative feedback loop to suppress NO production [24]. Although the level of NO recovered to control levels at 48 hpe, NO modulation caused by TD_49_ exposure might disturb a variety of immune homeostatic and other physiological processes.

ACP is a vital hydrolytic enzyme in phagocytic lysosomes [25,26], which participates in degradation of foreign proteins, carbohydrates, and lipids [27]. Our data revealed that the ACP activity was enhanced by exposure to TD_49_ at various time intervals. The present results are consistent with a previous study reported by Jing et al. [28], in which ACP activity in *Pinctada fucata* was increased in response to copper exposure. A previous study by our group found that ACP activity was increased by PA treatment. Increased ACP activity should enable phagocytes to destroy and clear pathogens more effectively, conferring resistance to long-term pathogen invasion [4]. Therefore, our results from this study suggest that ACP activity was increased to prevent scallops from TD_49_ invasion. The enzyme ALP has also been found in multiple bivalves; it participates in the degradation and breakdown of invading exogenous substances [17,29]. According to the present results, we also found that ACP might participate in cleaning the lower concentration of TD_49_ invading, while with the extension of exposure time, the higher concentration of TD_49_ showed better inhibition of ACP activity than the lower concentration of TD_49_ up to 48 hpe. It could be speculated that prolonged exposure to a high concentration of TD_49_ could induce immune fatigue in bay scallop. Similarly, our experiments also revealed that low and medium concentrations of TD_49_ (up to 48 hpe) induced hemolymph ALP activity. This is consistent with the previous study that ALP activity in the digestive gland of pearl oysters (*P. fucata*) exposed to copper was increased at 24 and 48 hpe [28]. Moreover, at the highest concentration of TD_49_, ALP activity was increased at 3–24 hpe, then decreased below that of control at 48 hpe. This is consistent with the previous study of copper exposure, which showed inhibition of ALP activity in the digestive gland of pearl oysters up to 72 hpe [28]. Meanwhile, our previous study also found a similar phenomenon in that low concentrations (20 mg/L) of PA significantly increased ALP activity in bay scallops from 3 to 24 hpe, with the higher concentration (40 mg/L) of PA enhancing activity at 3 and 6 hpe. This was followed by a significant decrease at 24 hpe, after which the values increased to normal at 48 hpe [5]. Therefore, the current study suggests that ALP activity might be increased to participate in eliminating lower and short time TD_49_ invading, while the high concentration and longer time of TD_49_ exposure up to 48 hpe could induce inhibition of ALP activity, restraining immune function.

Tomanek [17] revealed that multiple stress-induced proteins, including molecular chaperones, might be induced by cellular stress. Our experiments demonstrated that total protein levels were higher after TD_49_ exposure at each time point. These data are in accord with those reported by Hannam et al., who showed a significant increase in plasma protein levels in arctic scallops following dispersed oil exposure [17], and in the scallop *Pecten maximus* following phenanthrene exposure [18]. They also found that total protein levels were significantly elevated following PA exposure at 3–48 hpe. Hence, we speculate that TD_49_ exposure might cause cytolysis of hemocytes, releasing their contents into the hemolymph.

Immune recognition, which discriminates non-self from self, plays an essential role in initiating the immune response [11]. Immune responses begin when specialized, soluble, or cell-bound pattern recognition receptors (PRRs) recognize their major targets, called pathogen-associated molecular patterns (PAMPs) [11,30]. To date, some groups of distinct PRRs in scallops have been identified, which include peptidoglycan recognition proteins (PGRPs) [31] and C-type lectins (CTLs) [32]. PGRPs specifically bind to peptidoglycan, a unique cell wall component of nearly all bacteria but not present in eukaryotic cells. PGRPs not only act as PRRs, but also serve as scavengers in a battery of responses against invaders [11]. Our data revealed that the *PGRP* expression was inhibited momently after exposure to all three concentrations of TD_49_ at 3 hpe, and subsequently obviously enhanced with the exposure time increasing from 6–48 hpe. This result showed dependence of time and dose within a certain range, especially at 6 hpe, when the *PGRP* expression in the low concentration group returned to normal level compared to control, with a maximum expression at the high concentration group. This result is in agreement with a previous study that showed that the *PGRP* expression in bay scallop hemolymph was significantly increased by exposure to the algal toxin okadaic acid (OA). Combined, these results suggest that *PGRP* participates in eliminating TD_49_. The scallop CTLs possess multiple functions and play an essential role in host immune defense against invaders by recognizing and binding PAMPs on the surface of microbes, agglutinating them. They endow scallop hemocytes with the ability to phagocytize and encapsulate invaders [11]. We found that scallops exposed to a low concentration of TD_49_ had increased expression at 3, 12, and 24 hpe, showing a limiting and transitory resistance to low concentrations of TD_49_ exposure, while medium and high concentrations of TD_49_ had decreased *CLT-6* expression at all intervals, which implies dose dependence. In an earlier study, it was reported that scallops treated with a lower concentration (40 mg/L) of PA had higher *CLT-6* expression at 6–12 hpe, while a high concentration (80 mg/L) of PA had significantly lower *CLT-6* expression at 3, 6, 24, and 48 hpe [4]. We also found that scallops exposed to OA had decreased *CLT-6* expression at each time point [33]. These results indicate that higher concentrations of TD_49_ exposure could inhibit *CLT-6* expression, which may subsequently decrease the phagocytosis and encapsulation activities of hemocytes.

Metallothioneins (MTs), a superfamily of cysteine-rich proteins, perform multiple functions, including maintaining homeostasis of essential metal concentrations, detoxification of toxic metals, and scavenging of oxyradicals [11,34]. In this study, we observed that bay scallops treated with 0.34 μM of TD_49_ showed significantly higher *MT* expression than control at 3–24 hpe, significantly decreasing at 48 hpe, suggesting that low concentrations and transitory (less than 48 hpe) TD_49_ exposure induce the *MT* expression for scavenging TD_49_. This phenomenon is similar to that observed in the previous study, with the low concentration of PA elevating *MT* mRNA expression; however, the high concentration of PA obviously inhibited it in the hemolymph of bay scallop [4], which is consistent with the current results that the higher concentrations of TD_49_ inhibit the *CLT-6* expression at all time points. In summary, metallothioneins might participate in eliminating TD_49_ at low concentrations, but higher concentrations of TD_49_ may suppress *MT* expression, suggesting high time and concentration dependence.

In conclusion, we revealed a shift in bay scallop immune responses induced by exposure to the algaecide TD_49_. The significant changes found in several of the immune-related parameters we measured (SOD, ALP, ACP, NO, and the total protein), as well as in the expression of immune response-related genes (*PGRP*, *Cu/Zn-SOD*, *MT*, *PrxV*, and *CLT-6*), suggest that TD_49_ can affect immune function that normally mitigates damage in scallops experiencing xenobiotic invasion. However, the physiological responses and immunotoxic mechanisms altered by TD_49_ are complicated. It might act as an environmental disruptor and affect the immune system, even after acute toxicity assessment via the new ISO standard toxicity assessment method. Therefore, our present investigation, in addition to earlier research, further verifies that the use of TD_49_ as an algaecide poses a potential risk to scallop production. Furthermore, the results of this study highlight the necessity to understand the effects of a novel algaecide on other non-target species, especially economically important marine species, to develop environmentally friendly algaecides. However, the synergistic effect on the immune system or physical responses of bay scallops induced by TD_49_ needs to be further studied.

## 4. Materials and Methods 

### 4.1. Thiazolidinedione Derivative TD_49_

TD_49_ was synthesized as described by Kim et al. [2] and kindly provided by Hoon Cho, Department of Polymer Science and Engineering, Chosun University. It was stored at 4 °C. 

### 4.2. Bay Scallop and Hemolymph Sample Collection

Bay scallops (mean weight 46.6 ± 3.9 g; shell length 60–70 mm) were purchased from the Noryangjin wholesale fishery market in Seoul, South Korea. Before the experiments, the scallops were stored in lantern nets suspended in an 800-litre container closed system supplied with filtered and aerated seawater for two weeks to adapt to laboratory conditions (temperature: 10 ± 1 °C; salinity: 30 ± 0.1‰; constant aeration using air stones). Instant Algae® Shellfish Diet was fed to scallops with 1.2 × 10^10^ algal cells per scallop, with changing 50% seawater daily before the treatment trial. This present study was approved by the Animal Care and Use Committee of Nanjing Agricultural University (Nanjing, China) (permit number: SYXK (Su) 2017–0007).

Three hundred and sixty bay scallops were randomly divided into four groups: Control group and three experimental groups (DA exposure). Each group comprised 30 scallops with 3 replicates. TD_49_ was dissolved in 1 mL of dimethyl sulfoxide (DMSO) and maintained the final concentrations in TD treatment groups at 0.34, 0.58, and 0.68 μM. The bay scallops in the control group were treated with an equal volume of DMSO vehicle. Three scallops from each replicate treatment group were randomly sampled at 3, 6, 12, 24, and 48 hpe. Two millilitres of hemolymph was collected from each adductor muscle using a 1 mL sterile syringe fitted with a 22-gauge needle within 1 min of removing each scallop from the tank. Three scallops per group were used as triplicates. The hemolymph sampled from each group was divided into two parts: One part (100 µL) was used to extract RNA, and the other part was centrifuged for 3 min at 750 × *g* and stored at −80 °C to measure the humoral immune parameters.

### 4.3. Measurement of Enzymatic Activity in Hemolymph

SOD activity was measured by a commercial SOD activity assay kit (Nanjing Jiancheng Bioengineering Institute, China) following the manufacturer’s instructions. The reduction rate of cytochrome c by O_2_ was monitored spectrophotometrically at 550 nm and 25 °C using a Shimadzu UV-240 spectrophotometer. The xanthine–xanthine oxidase system was utilized as the source of O_2_^−^. One unit of SOD was defined as the amount of enzyme needed to inhibit the rate of cytochrome c by 50%.

ACP and ALP activity in the hemolymph were quantified using assay kits (Nanjing Jiancheng Bioengineering Institute, China). The concentration of enzymes in 100 mL hemolymph, which yielded 1 mg nitrophenol within 30 min at 37 °C, was defined as a unit of ACP activity. One microgram of phenol was released per 100 mL hemolymph, corresponding to one unit of ALP activity.

NO levels were determined using the nitrate reductase assay using an assay kit (Nanjing Jiancheng Bioengineering Institute, China). NO was oxidized to nitrate and nitrite by oxygen and then reacted with the chromogenic agent to generate azo dyes, which could be quantified using a spectrophotometer.

Total protein concentration in scallop hemolymph was determined using a kit (Nanjing Jiancheng Bioengineering Institute, Jiangsu, China), following the manufacturer’s instructions.

### 4.4. Quantitative Measurements of Gene Expression

Total RNA was extracted from hemolymph using RNAiso Plus (TaKaRa Bio, Ostu, Japan). The quality and purity of RNA were assessed spectrophotometrically by the ratio of absorbance at 260 and 280 nm. Only RNA samples with A260/A280 ratios between 1.8 and 2.0 were used for subsequent analyses. Genomic DNA contamination was removed using DNase I (Promega, Madison, WI, USA). The cDNA was then synthesized using the PrimeScript^TM^ RT Reagent Kit (TaKaRa Bio, Ostu, Japan) as per the manufacturer’s instructions, then stored at −80 °C for use in real-time quantitative PCR (RT-qPCR).

qPCR was performed using SYBR Premix Ex Taq™ Kits (TaKaRa Bio) on a RT-PCR Detection System (Qiagen, Hilden, Germany). Gene expression was standardized by *β-actin*. The sequences of the PCR primers of genes (*CTL-6*, *PGRP*, *PrxV*, *MT*, and *Cu/Zn-SOD*) used for qPCR are listed in Table 1. The reaction mixture included 10 μl SYBR Premix Ex Taq^TM^, 1 μl of the forward and reverse primers (10 mM), and 1 μl cDNA. Ultra-pure water was then added to the reaction to make up the final volume to 20 μl. The reaction conditions were as follows: 95 °C for 10 min, followed by 40 cycles of 95 °C for 45 s, 56 °C for 45 s, and 72 °C for 30 s. After amplification, a melting curve analysis was conducted to test for the possibility of nonspecific amplification or primer-dimer formation [35]. Relative mRNA levels were calculated by the 2−ΔΔCt method [36]. In all the cases, PCRs were carried out in triplicate.

### 4.5. Statistical Analysis

The data were tested for normality and homogeneity of variance, using the Kolmogorov–Smirnov and Cochran’s tests. Data were analyzed using one-way analysis of variance (ANOVA). The data are presented as mean ± standard deviation (SD) values. The differences were determined using the Duncan test in SPSS 19.0 software (IBM Corp., Armonk, NY, USA) with *p* < 0.05 indicating statistical significance.

## Figures and Tables

**Table 1:** Primers used for the analysis of mRNA expression by qRT-PCR.

Genes	Primer Sequence	Accession No.
*β-actin*	F: 5′CAAACAGCAGCCTCCTCGTCA 3′	AY335441
	R: 5′CTGGGCACCTGAACCTTTCGTT 3′	
*PrxV*	F: 5′AATCAAGGAGCGGCTGGCA 3′	HM461987
	R: 5′TCAACTTCTCAATCTTCCCGTCAT 3′	
*CTL-6*	F: 5′CAGTTGCTACAGGGTTCG 3′	GQ202279
	R: 5′GGGCGTTATCTGGCTCAT 3′	
*MT*	F: 5′AACTTGCTGTAGTGGGAATG 3′	EU734181
	R: 5′AGGCTGGAAACTGCTGTGGT 3′	
*PGRP*	F: 5′GGGCAAGTGTATGAGGGAAGAG 3′	AY437875
	R: 5′TCCGATGAAGGAGACAGCGTAG 3′	
*Cu/ZnSOD*	F: 5′GTATTGAAAGGTGATTCGGAGG 3 ′	EU563958
	R: 5′ATGCACATGAAAGCCATGTAGG 3 ′	
